# P05.09. "Without it, it would have been much worse": a mixed-method evaluation of clinical reflective practice in integrative care education

**DOI:** 10.1186/1472-6882-12-S1-P369

**Published:** 2012-06-12

**Authors:** G Lutz, F Edelhäuser, C Scheffer, D Tauschel, M Neumann

**Affiliations:** 1 University of Witten-Herdecke, Witten, Germany

## Purpose

Reflective abilities are seen as helpful in improving patient-centered care and personal professional development and are therefore advocated especially in integrative care education, since integrative medicine involves personalized care. The aim of our study is twofold: To present (1) a qualitative evaluation of clinical students on a newly developed clinical reflective practice (CRP) format; and (2) a quantitative study with self-assessed pre- and post measurements of distress and personal reflexivity.

## Methods

The CRP took place on a clinical integrative education ward, where under close clinical supervision groups of 3-5 students took care of patients. Every two weeks they reflected on their experiences with a supervisor. Sixteen individual and focus group interviews were conducted and analyzed according to thematic content analysis. Pre- and post stress-measurements were assessed in every CRP using a 10-point scale using the Distress-Thermometer. Students’ reflexivity was assessed with the GRAS-measure and was also measured before the first and after the last CRP.

## Results

Major results observed were that 100% of students found the reflective experience helpful to survive the vortex of events when immersing into the responsible care for patients. They reported subjectively significant effects on themselves, on team work and on the patient, e.g. stress-reduction, positive change in perception of pitfalls, comprehensibility of self and other, experience of meaning, options to act, capacity to provide feedback, tolerance for ambiguity and complexity, openness, improved ability for conflict management, willingness to help and feedback culture. Students’ distress-levels seem to diminish in a significant way through CRP.

## Conclusion

This CRP approach could be a tool to develop more patient-centered, individualized care and thus lead to more satisfying outcomes for students and patients in integrative care.

